# A Rapid Detection Method for Morphological Characteristics of Biological Cells Based on Phase Imaging

**DOI:** 10.1155/2018/4651639

**Published:** 2018-04-11

**Authors:** Wen-Bo Tang, Ying Ji, Ming-Ming Zhang, Zhi-Ya Chen, Yuan-Yuan Xu, Ya-Wei Wang

**Affiliations:** ^1^Faculty of Science, Jiangsu University, Zhenjiang 212013, China; ^2^School of Mechanical Engineering, Jiangsu University, Zhenjiang 212013, China

## Abstract

This paper presents a rapid label-free method for the identification of morphological characteristics of biological cells. Based on quantitative phase microscopy as well as the connotation of phase value, the gradient operator of phase and the associated analytic processing are employed to determine the edge of different parts of the samples. A heterogeneous biological cell model is established by simulation to show the mechanism of this method and a polystyrene bead is selected as a sample to confirm its validity by optical experiment. The result agrees well with the actual situation and this approach is proved to have good antinoise ability. Furthermore, a neutrophil is investigated by this method. Based on the optical experiment and the related analysis, the basic structure characteristics of the cell are obtained. It is indicated that the method presented in this paper could be applied to rapid identification and classification of living cells.

## 1. Introduction

Biological cell is the basic unit of the organism and it plays an important role in life activities. It has different functions as well as the associated morphological structures: that is, the status of a cell is closely related to its shape and structure. Therefore, the research on the morphological characteristics of biological cells has been widely concerned in medical applications to diagnose disease as well as analyzing the related mechanism.

Since the biological cells are colorless and transparent, ordinary optical imaging techniques do not work. To detect and demonstrate the morphology of cells, many techniques have been proposed, such as immunofluorescence technique, scanning electron microscope, and Hematoxylin-Eosin (HE) staining [1–3]. In these cases, routine dyeing method is widely used, which needs complicated operating steps. Furthermore, one of the consequences of staining is that the activity of the cell will be damaged. As a noninvasive microscopy, quantitative phase microscopy (QPM) is significantly more convenient than staining [4]. It achieves the transparent sample imaging by recording the phase shift induced by the sample, without being damaged by labeled drugs or phototoxicity. The label-free operation means that QPM is especially applicable to live cells imaging. It has attracted more and more attention and has obtained many encouraging results [5–8], such as spatial light interference microscopy (SLIM), which can provide nanoscale information about biological structures [8]. However, QPM for substructure identification of nucleated cells is still an open problem since the physical thickness information of the sample is coupled with the refractive index in the phase value. Because the refractive index is exactly the intrinsic property of a biological cell, some decouple methods have been proposed to get the refractive index distribution to determine the sample morphology, such as dual wavelength technique and confocal technique [9–11]. Though the average refractive index of each part of the sample can be obtained from these approaches, the addition of operation procedures results in more processing time and cost. Besides that, it may lead to more measurement errors and noise. Thus, these methods are not suitable for rapid identification of sample characteristics. Inspired by morphological edge detection and combined with the essential feature of phase function, a rapid detection method is proposed in this paper, which is easy to operate and has good antinoise ability.

## 2. The Gradient Analysis Method

**Figure 1:**
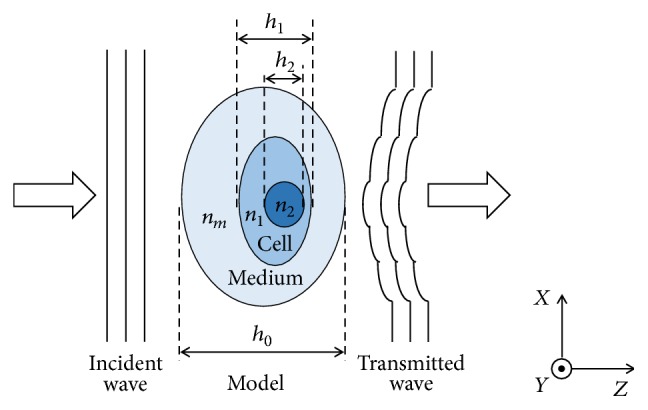
Two-dimension model of a multimedium sample.

Gradient operator is a common edge detection operator. Considering that the phase shift of the transmitted field equals the line integral of the refractive index along the path of beam propagation, the refractive index variation of the sample is sensitive to the gradient of the phase. That is, the gradient of the phase distribution could be employed here to determine the edge of the substructure of the sample. To explain the mechanism, a transparent multimedium model is shown in Figure 1. Here, we assume the physical thickness of the cell body and that of cell nucleus in horizontal direction are *h*_1_ and *h*_2_, respectively. The refractive indexes of the cell nucleus and cytoplasm are defined as *n*_2_ and *n*_1_, respectively. A parallel light is supposed to transmit through such a model along *Z*-axis. To be specific, the light goes through the surrounding medium, cytoplasm, and cell nucleus in sequence and then passes through cytoplasm and surrounding medium again. Thus, the values of refractive indexes of the sample have the related alternation; that is, it increases from *n*_*m*_ to *n*_1_ and then continues to increase to *n*_2_ and then decreases to *n*_1_, until it decreases to *n*_*m*_. On the cross-section, that is, the imaging plane, the total phase shift induced by the cell can be described as

(1) $$\begin{array}{c} \varphi \left(\;x,y\;\right)=\frac{2\pi }{\lambda }\overset{h\left(x,y\right)}{\underset{0}{\int }}\left[\;n_{c}^{z}\left(\;x,y,z\;\right)-n_{m}\;\right]dz. \end{array}$$

Here *λ* is the wavelength of the light,  *h*  is the local thickness of the cell, and *n*_*m*_ is the refractive indexes of the surrounding medium. The quantity *n*_*c*_^*z*^ is the refractive index of cellular material, which is generally an inhomogeneous function in all three dimensions [12]. In general, *n*_*c*_^*z*^ can be represented by an average refractive index *n*_*c*_. So (1) can be rewritten as (2) in terms of axial average refractive index of different parts in this sample.

(2) $$\begin{array}{c} \varphi \left(\;x,y\;\right)=\frac{2\pi }{\lambda }\left[\;n_{c}\left(\;x,y\;\right)-n_{m}\;\right]h\left(\;x,y\;\right). \end{array}$$

From (2), the axial thicknesses and the associated refractive index information of the sample are coupled as it shows. Though the substructure of the transparent sample could not be presented directly from the quantitative phase information, the value of the phase shift is proportional to the axial thickness in one region which has the same refractive index. That is, in different regions, the change rate of the value of phase shift is also different. Based on this property, the gradient of the phase shift is introduced to analyze the structure of the sample.

**Figure 2:**
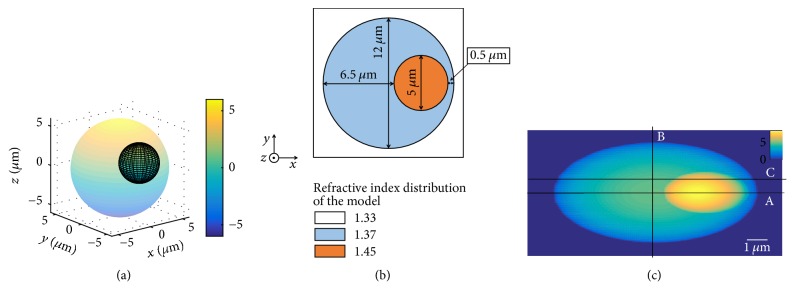
(a) 3D eccentric sphere model. (b) The projection in the *z*-axis direction of eccentric sphere model. (c) Optical phase distribution of the eccentric sphere model.

Before the investigation of a real cell, a 3D eccentric sphere model is established to demonstrate the detection procedure, which is shown in Figure 2(a). The projection in the *Z*-axis direction of the model is shown in Figure 2(b), where the big sphere and the small sphere could, respectively, imitate the cytoplasm and the nucleus of the heterogeneous cell. According to the cell physiological parameters, the radius of the big sphere  *R*_1_  is set to 6.0 *μ*m, and the radius of the small one is *R*_2_ = 2.5 *μ*m. The center of the big sphere and the small one is set at (0,0, 0) and (3, 0,0), respectively. In *x*-axis, the distance between the borderline of the small sphere and the big one is set to 6.5 *μ*m and 0.5 *μ*m, respectively (shown in Figure 2(b)). The size of the recorded plane is 15 × 15 *μ*m^2^, and the recorded plane has 255 × 255 pixels. Thus, the spacing between two adjacent pixels on this plane is 15 *μ*m/255. The refractive index of the nucleus and cytoplasm in this model are set to *n*_2_ = 1.45 and *n*_1_ = 1.37. The refractive index of the surrounding medium is *n*_*m*_ = 1.33 and the wavelength of the light source is 632.8 nm. The light is assumed to transmit along *Z*-axis. According to (2), the distribution of the two-dimensional phase of the model is expressed as (3) specifically. And the associated optical phase distribution of this model is shown in Figure 2(c).

(3) φx,y=2πλn1−nm×2×R12−x2−y2+n2−n1×2×R12−x−32−y2.

**Figure 3:**
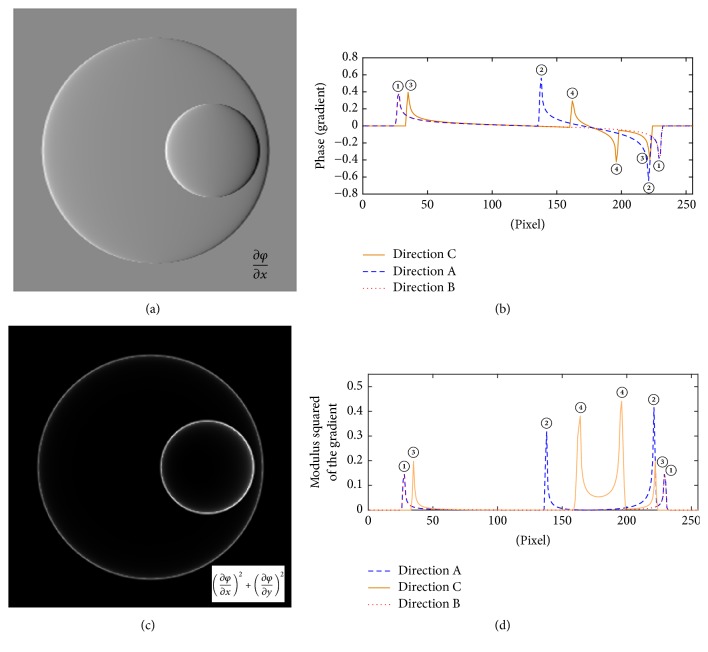
(a) The lateral phase gradient distribution of eccentric sphere model. (b) The phase gradient curves in different directions of eccentric sphere model. (c) The modulus squared lateral phase gradient distribution of eccentric sphere model. (d) The modulus squared phase gradient curves in different directions of eccentric sphere model.

From Figure 2(c), one could see that though a phase image can indicate the morphological characteristics of the model to some extent, it cannot be looked at as the morphological distribution directly, especially for its substructure. It could not be sure which factor induces the phase shift, whether the thickness or the refractive index of the medium. According to the gradient analysis mentioned above, the gradient of the phase in *x*-direction is calculated by (4), and its distribution is shown in Figure 3(a).

(4) ∂φx,y∂x=2πλn1−nm×−2xR12−x2−y2+n2−n1×−2xR12−x−32−y2.

Obviously, Figure 3(a) demonstrates a rounded shape which indicates the characteristics of the sample. The size and position of the model and its nuclei are displayed clearly. But it has the “shadow artifact” which is caused by the first-order derivative changing sign across an edge [13]. In order to remove the “shadow artifact” in Figure 3(a), the modulus squared of the gradient is calculated in Figure 3(c). The graph is found to be exactly clearer to reflect the axial boundary projection of the sample. Furthermore, to demonstrate the quantitative information which is hidden in the phase gradient, we take the gradient distribution curve, that is, the 1D gradient distribution in one direction, to visually express the spatial dimension information of the sample. For example, the phase gradient distribution at the transverse section which refers to *y* = 0 is described as

(5) ∂φx,0∂x=4πλn1−nm×−xR12−x2+4πλn2−n1×−xR12−x−32=C1×−xR12−x2+C2×−xR12−x−32,

where *C*_1_ = (4*π*/*λ*)(*n*_1_ − *n*_*m*_) and *C*_2_ = (4*π*/*λ*)(*n*_2_ − *n*_1_) are definite constants. It should be noted that, when *x* = ±*R*_1_ or *x* = 3 ± *R*_2_, that is, the boundary of eccentric sphere model, ∂*φ*(*x*, 0)/∂*x* does not make sense according to (5) in theory. While according to forward difference operation, the phase gradient ∂*φ*(*x*, 0)/∂*x* is calculated as a relative large value at these locations, which appears as a jump in gradient distribution curves. And the variation value of the refractive index is related to the change value of refractive index between adjacent medium at each side of the interfaces.

In order to investigate the quantitative substructure information of the model more comprehensively with gradient distribution curve, the variation curves of phase gradient in different directions are calculated, that is, directions A, B, and C shown in Figure 2(c), which are presented in Figure 3(b). Take the variation in direction A as an example, the jump points come in pairs because the variation of the refractive index is in pairs along the light which goes through the sample. The positions of these jump points are approximate to (28, 0), (138, 0), (221, 0), and (229, 0). Similarly, in the vertical curve of the horizontal gradient (shown in Direction B), the calculation direction does not pass through the nucleus, so there are two fluctuations and the positions of these jump points are approximate to (28, 0) and (229, 0). In Direction C, there are also two pairs of symmetrical positive and negative jumps, where coordinate values are approximate to (35,0), (162,0), (196,0), and (222,0). This direction does not through the center of the sphere, which could be demonstrated by the calculated distance between the jump points.

For comparison purpose, the similar variation curves of the modulus squared gradient in the relevant directions (A, B, and C) are also calculated (shown in Figure 3(d)). It is found that, in all three directions, the mutation positions which appear in the modulus squared curves coincide with the case of gradient curves and the gradient curves (shown in Figure 3(b)) can reflect the change trend of the refractive index more significant along incidence directions.

In this simulation, this fluctuation position corresponds to the boundary points of each medium, including between cytoplasm and cell nucleus. That is, these fluctuations are related to the variation of the refractive index inside the cell. Thus, the distance between the pair of points, such as points ①-①, ②-②, ③-③, and ④-④, indicates the size of each fraction of the sample. By conversion, the specific distances between different pairs of points in Figures 3(b) and 3(d) are listed in Table 1 in detail.

It could be seen that the result agrees well with the morphology of the model shown in Figure 2(a). For the nuclei and the cytoplasm, the maximum error of the diameter value is calculated at 0.177 *μ*m, and the average relative error is 2.5%. Moreover, it could be seen that the fluctuations on the phase gradient curve are sensitive to the direction. These fluctuations in the orthogonal directions can reflect the structural information of the sample.

**Figure 4:**
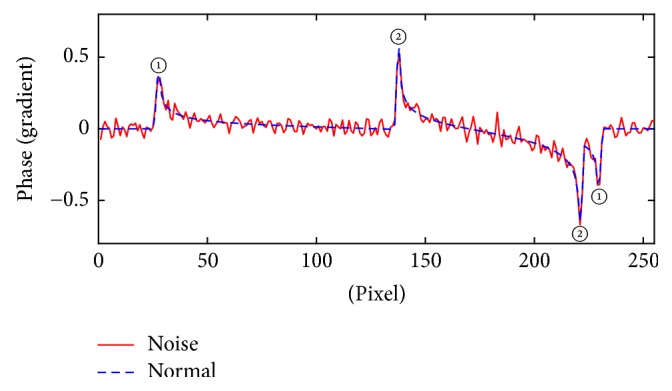
The comparison of phase gradient curve of the sphere model in Direction A with Gaussian noise.

From the above analysis, the 2D modulus squared lateral phase gradient distribution and the 1D phase gradient curves are suggested to be appropriate for the determination of morphological characteristics. Besides, the influence of the noise is taken into account as well. The white Gaussian noise is introduced to the phase images, which is set at 35 dB signal-noise ratio. The phase gradient curve in Direction A is taken as an example to discuss the effect (shown in Figure 4). The positions of these jump points are approximate to (28,0), (138,0), (221,0), and (229,0). The result is similar to the case without noise, since the jump of phase gradient is not affected by noise, which means the method has good noise immunity.

## 3. Experiment Result and Analysis

**Figure 5:**
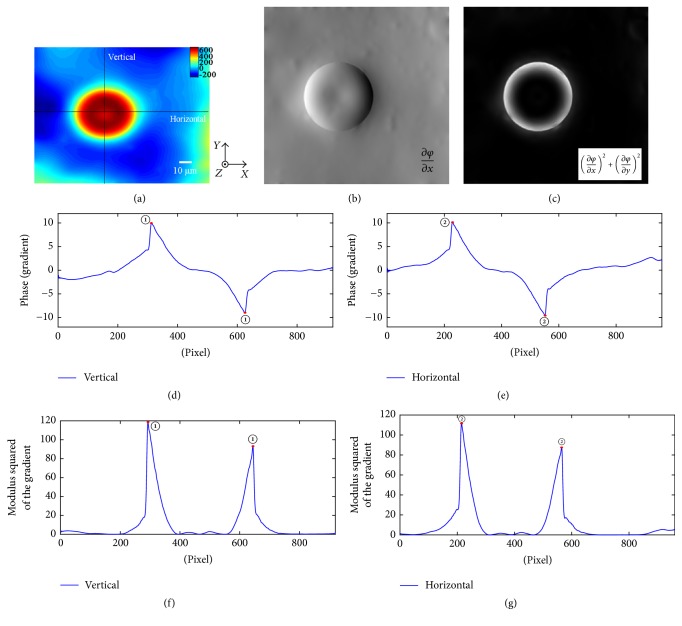
(a) The phase-rendered image of the polystyrene bead. (b) The lateral phase gradient distribution of the polystyrene bead. (c) The modulus squared lateral phase gradient distribution of the polystyrene bead. ((d) and (e)) The vertical and the horizontal phase gradient curves of the polystyrene bead, respectively. ((f) and (g)) The vertical and the horizontal modulus squared curves of the polystyrene bead, respectively.

Next, optical experiment is implemented to prove this method. In the experiment, the Bio-Phase system was used to image polystyrene beads with an average diameter of 50 *μ*m. Bio-Phase is a new imaging device based on digital wave-front detection technology that performs real time phase imaging [14]. And the related parameters are set as follows: wavelength of the light source is 633 nm and magnification of microscope objective lens is 50. Under the experiment condition, the lateral resolution of Bio-Phase system is 0.4 *μ*m, and the axial resolution is 1.1 *μ*m. The phase distribution of the polystyrene bead can be observed as Figure 5(a). For digital phase imaging, the height and color of a point on the image correspond to the optical thickness of the sample and depend on the relative refractive index as well. In the phase distribution diagram, the depression in phase center is caused by the reflection of polystyrene bead.

According to the above method, the phase gradient in *x*-direction is calculated in Figure 5(b). Similarly, in order to remove the “shadow artifact” in Figure 5(b), the modulus squared of the gradient is calculated in Figure 5(c). For simple and quick analysis, the gradient curves at two orthogonal directions which pass through the phase center are selected.

The vertical phase gradient curve as well as the horizontal one is presented in Figures 5(d) and 5(e), respectively. In both figures, it could be seen that there is only one large fluctuation, which are marked as the pair of points ① and ② at the curve. The specific distance and its error between different points can be obtained by conversion and are described in Table 2 in detail.

Similarly, the vertical and the horizontal modulus squared curves are calculated and presented in Figures 5(f) and 5(g), respectively. By comparison, it is found that, in these two directions, the mutation positions which appear in the modulus squared curves coincide with the ones in the phase gradient curves. From the above analysis, it could be seen that the result is consistent with the actual situation of the sample. That is, the validity and advantage of the analysis, based on modulus squared of phase gradient and phase gradient curves, for morphological identification are also proved by experiment.

**Figure 6:**
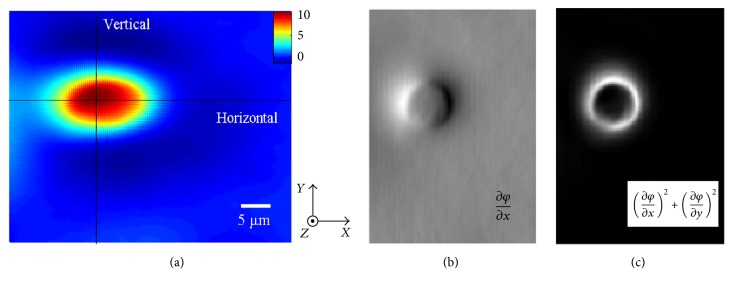
(a) The phase-rendered image of the neutrophil sample. (b) The lateral phase gradient distribution of the neutrophil sample. (c) The modulus squared lateral phase gradient distribution of the neutrophil sample.

Furthermore, a cell sample, that is, a neutrophil, is investigated to get the morphological structures information. Such a sample plays a very important role in the immune system, which can change its shape to pass through the vascular endothelial cells and arrives at the infected site to have phagocytosis and scavenging effect on the invasion of pathogens. To detect the cell's shape, the phase distribution of the sample can be observed as Figure 6(a) which is obtained by the Bio-Phase system. The experimental condition is set as above. The phase gradient as well as the modulus squared of the gradient are calculated, which are shown in Figures 6(b) and 6(c), respectively.

**Figure 7:**
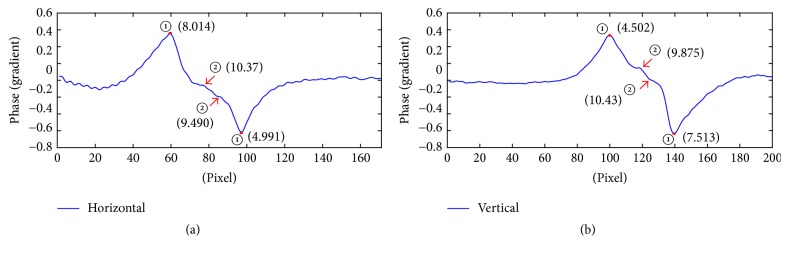
The horizontal and the vertical phase gradient curves of Figure 6(a), respectively (the number in brackets is the phase value at that point).

The phase gradient curves of this sample at two orthogonal directions which pass through the phase center are shown in Figures 7(a) and 7(b).

In Figures 7(a) and 7(b), it could be seen that there are two large fluctuations, which are marked as the pair of points ① at the curves. Besides these two large fluctuations, some other small fluctuations can be observed also. The small fluctuations outside the pair of points ① seem to be irregular, which may be caused by noise or other factors. On the curves in Figures 7(a) and (b), a pair of small fluctuations between points ① is marked as points ②. Points ② have some similarities in both figures, including their positions and the distance between them. In particular, the amplitudes of these two fluctuations at orthogonal directions are similar, which means the variations of the phase are alike at these two directions in the corresponding region. As for the positions of these jump points, the coordinate values of the pair of points ① and ② in Figure 7(a) are approximate to (60,0), (76,0), and (84,0), (97,0), and the corresponding phase values are 8.014, 10.37, 9.490, and 4.991, respectively. In Figure 7(b), the positions of these jump points are approximate to (100,0), (118,0) and (125,0), (139,0); the corresponding phase values are 4.502, 9.875, 10.43, and 7.513, respectively. The distances between different points can be obtained by conversion and described in Table 3 in detail.

As the analysis in the previous section, to reveal the mechanism of this variety as well as the substructure characteristics of the sample, we still assume the light transmits along *Z*-axis. Based on numerical calculation as well as the analysis above, the pair of symmetrical larger jumps observed clearly in Figure 7 is supposed to be related to the difference of refractive index between the cytoplasm and the surrounding medium. It is worth noting that the curve outside the points ① is rough. It may be caused by noise or some impurities in the surrounding medium. That is, points ① are at the interface between the surrounding medium and cytoplasm. Combined with the information shown in Table 3, it could be observed that the distance between points ①-① at the orthogonal directions are approximately equal, which indicates that the whole sample is nearly a sphere and its particle size is about 15 *μ*m. Moreover, a pair of similar small fluctuations present at both orthogonal directions means the existence of a nucleus. The values of the distance between points ②-② at these two directions are similar too, which shows that the cell nucleus appears spherical also and its position might be found off-center. The particle size of the nucleus is close to 3 *μ*m.

Based on the above analysis and calculation, the basic structure characteristics of the sample could be identified rapidly without any labeling technique and the result agrees well with the objective facts. It should be noted that though the result presented in this paper is not detailed enough compared with 3D reconstruction, it could be applied to rapid preliminary identification due to the simple operation.

## 4. Conclusion and Perspective

A rapid method based on phase imaging is employed to analyze the morphological characteristics of the biological cells in this paper. A nucleated cell model is discussed first by simulation. Based on the phase image as well as the property of the phase function, the substructure morphological character of the model is presented by simple gradient analysis of the phase value. Then this method is applied to a polystyrene bead by experiment. And this method is proved to be valid by the results both from the simulation and the experiment. Furthermore, the morphology of a neutrophil is explored. The substructure characteristics of the sample including axial size of the whole cell and the nucleus can be acquired. This paper presents a label-free approach for primary morphology identification of biological cells based on QPM with simple operation. To obtain 3D substructure information of the sample in detail, more phase images from different angles or layers are needed and the 3D morphological reconstruction based on QPM is our future work.

## Figures and Tables

**Table 1:** The distances between different points in the sphere model in different directions.

Distance between two points	Direction A		Direction B		Direction C	
	Measured value (*μ*m)	Set value (*μ*m)	Measured value (*μ*m)	Set value (*μ*m)	Measured value (*μ*m)	Set value (*μ*m)
①-①	11.823	12	11.823	12	∖	∖
②-②	4.882	5	∖	∖	∖	∖
①-② (left)	6.471	6.5	∖	∖	∖	∖
①-② (right)	0.471	0.5	∖	∖	∖	∖
③-③	∖	∖	∖	∖	11.000	∖
④-④	∖	∖	∖	∖	2.000	∖
③-④ (left)	∖	∖	∖	∖	7.471	∖
③-④ (right)	∖	∖	∖	∖	1.529	∖

**Table 2:** The distance and its error between different points in experiment.

Distance between two points and its relative error (*μ*m)	Actual value (*μ*m)	Measured value (*μ*m)	Relative error
①-①	50	50.6	1.2%
②-②	50	50.8	1.6%

**Table 3:** The distance between different points in the neutrophil sample.

Distance between two points	Horizontal	Vertical
①-①	14.8 *μ*m	15.6 *μ*m
②-②	3.2 *μ*m	2.8 *μ*m
①-② (left)	6.4 *μ*m	7.2 *μ*m
①-② (right)	5.2 *μ*m	5.6 *μ*m
